# Inferring the underlying multivariate structure from bivariate networks with highly correlated nodes

**DOI:** 10.1038/s41598-022-16296-y

**Published:** 2022-07-21

**Authors:** Philipp Loske, Bjoern O. Schelter

**Affiliations:** 1 Aberdeen Biomedical Imaging Center, University of Aberdeen, Foresterhill, Aberdeen, UK; 2 TauRx Therapeutics Ltd., Aberdeen, UK; 3 Institute for Complex Systems and Mathematical Biology, University of Aberdeen, Aberdeen, UK

**Keywords:** Applied mathematics, Statistics, Complex networks

## Abstract

Complex systems are often described mathematically as networks. Inferring the actual interactions from observed dynamics of the nodes of the networks is a challenging inverse task. It is crucial to distinguish direct and indirect interactions to allow for a robust identification of the underlying network. If strong and weak links are simultaneously present in the observed network, typical multivariate approaches to address this challenge fail. By means of correlation and partial correlation, we illustrate the challenges that arise and demonstrate how to overcome these. The challenge of strong and weak links translates into ill-conditioned matrices that need to be inverted to obtain the partial correlations, and therefore the correct network topology. Our novel procedure enables robust identification of multivariate network topologies in the presence of highly correlated processes. In applications, this is crucial to avoid erroneous conclusions about network structures and characteristics. Our novel approach applies to other types of interaction measures between processes in a network.

## Introduction

Complex systems can be found anywhere, from social interactions to power grids, from the description of a pandemic to the brain^1–4^. Complex systems can be described as a collection of individual objects interacting with each other, where the interaction plays a significant role in shaping the system’s essential functionality. Mathematically, the interacting objects are nodes, and their interactions are links in a network. Understanding these interactions by studying the network’s topology gives valuable insight into the fundamental properties and characteristics of the complex system.

Here, we define a network *G*(*V*, *L*) in the same way as Mader et al.^5^, with *N* nodes defined by $V=\{n_1, n_2, \dots , n_N\}$ and an ordered set of links $L\subset \{(n_i, n_j) \in V\times V\}$. The number of links $d_i$ connected to a node $n_i$ is called degree of the node^5^. For weighted networks, such as correlation networks, the weighted degree is separated into weighted positive $d_i^{+}$ and negative $d_i^{-}$ degrees as the sum over all positive $w_i^{+}$ and negative $w_i^{-}$ weighted links connected to $n_i$, respectively^6^. Networks can be represented in a $N\times N$ matrix *M* where the rows and columns correspond to the nodes of the network. These nodes typically represent or refer to directly measured features or variables of the system. *M*(*i*, *j*) is the link $w_{ij}$ connecting node $n_i$ with $n_j$. *M* is called the adjacency matrix and consists of ones and zeros for binary networks or weights $w_{ij}$ for weighted networks. For undirected networks, such as correlation networks, *M* is symmetric^7^.

Inferring the links of a network from observation can be a challenging task^8^. It is essential to have a reliable network structure that resembles the underlying network topology of the complex system. One standard measure to describe how objects are related is the correlation coefficient. The correlation coefficient provides a convenient and often applicable tool for constructing networks. However, it is a bivariate measure; hence it does not distinguish between direct and indirect links. If naively analysed, indirect links can lead to incorrect decisions about the inferred network and its topology^5,9–12^.

Instead of analysing the bivariate measure directly, it is often preferable to analyse the multivariate counterpart, which consists of only direct links between nodes^13^. The partial correlation coefficient, which is the corresponding multivariate measure for the correlation coefficient, measures the correlation between pairs of nodes by considering the influence of all other random variables that are part of the network. Strictly speaking, it is the correlation between these nodes’ processes; as it is evident from the context what is meant, this distinction is not made going forward unless needed for clarity. The partial correlation coefficient can be inferred from the correlation coefficient using matrix inversion^14^. A matrix inversion is only possible if the matrix representation of the network has full rank, i.e., the rows of the matrix are linearly independent. Matrix inversion becomes problematic numerically already when the matrix is ill-conditioned; this, for instance, is the case when components are highly correlated. The matrix representations of such networks are strictly speaking mathematically invertible; hence the corresponding multivariate network can be inferred in principle. However, the high correlations and therefore ill-conditioned matrix result in an inferred multivariate measure that is unstable and does not resemble the actual underlying network. Thus, the question arises of how to handle real-world networks with full rank but containing nodes with high correlations.

Here, we want to analyse how the multivariate measure is affected in the limit of highly correlated but linearly independent nodes. The assumption here is that features or variables representing the nodes form a network for which the matrix describing the interactions or links cannot be numerically inverted. This is a step that is often needed to translate bivariate measures into multivariate interacting measures. An example to demonstrate this is the correlation coefficient, quantifying the bivariate interaction, and its multivariate counterpart the partial correlation. We will use this as an example throughout the manuscript, but our approach is readily applicable to other linear interaction measures such as coherence and partial coherence^13,15^ as well as nonlinear interaction measures such as the phase synchronisation and partial phase synchronisation approach^16^. We propose a new method to reconstruct the multivariate network that reduces the dimensionality of the correlation matrix by automatically merging highly correlated nodes before inverting the matrix. We also test the robustness of this method against a direct inference of the multivariate measure in the presence of highly correlated nodes in a simulation study.

**Figure 1:**
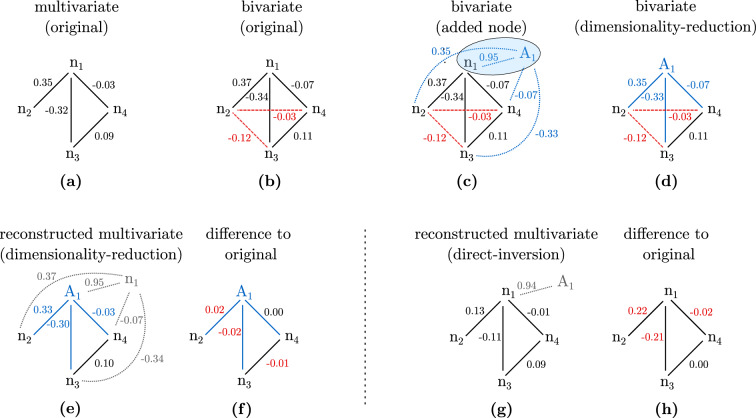
Overview over the various steps of our method and results: (**a**) A network consisting of only direct links. (**b**) The corresponding bivariate network consisting of direct as well as indirect links. Indirect links that are not present in the multivariate network are illustrated with a dashed red line. (**c**) Node $A_1$ is added to the network with a high correlation to node $n_1$, causing a small determinant of 0.07. (**d**) The dimensionality-reduction method merges nodes based on a threshold set to $T=0.1$ for the determinant, here node $n_1$ and $A_1$. The reduced bivariate network has a determinant of 0.77 and the algorithm stops merging nodes. (**e**) The reconstructed multivariate network by inverting the network in (**d**). The formerly removed node $n_1$ is added to the network again. (**f**) Difference to the original multivariate network. (**g**) Reconstructed multivariate network by directly inverting the network shown in (**c**) and (**h**) the difference to the original multivariate network.

This is a different approach to regularisation of the matrix based approaches^17,18^, which provides full inversion in an unreduced setting. As we will show, our approach gives the correct partial correlation structure, albeit for the reduced network, while the regularisation approach only gives an approximation to the partial correlation matrix. It stands to reason that the more the original matrix is ill-conditioned, the less accurate the approximation. In the supplementary information we show, using ridge regression and exemplified by the network shown in Fig. 1, that the direct-inversion and dimensionality-reduction methods perform better than the regularisation method.

## Materials and methods

This section describes the method to reconstruct the multivariate measure in the presence of highly correlated nodes using a dimensionality-reduction method. The bivariate measure is represented by the correlation coefficients, and the multivariate by the partial correlation coefficients^5^.

In short, the method analyses the determinant of the correlation matrix. If the determinant is smaller than a set threshold, the procedure removes nodes iteratively until the determinant of the reduced matrix is greater than the threshold. The removal of nodes can be interpreted as merging two nodes that provide similar information into a single node. The reduced matrix is inverted to reconstruct the reduced multivariate network. Previously removed nodes are added again to the network.

The described algorithm is tested in a simulation study against the direct inversion without dimensionality reduction. To be able to compare both algorithms, the actual multivariate networks need to be known.

The algorithm for the reconstruction of bivariate networks based on actual multivariate networks is described in Sec. “Construction of multi- and bivariate networks”; the simulation setup is discussed in Sec. “Simulation setup”.

### Method steps

Let *M* be a $N \times N$ matrix representation of a bivariate measure, such as the correlation matrix $\rho$, with *N* nodes. Let $M_{i,i}$ be the $(N-1)\times (N-1)$ matrix, derived from *M* by removing the *i*-th node from the network, i.e., removing the *i*-th row and column.

The matrix *M* is passed on to the method together with a real valued threshold *T* for the determinant. If the determinant of *M* is smaller than the threshold *T*, it performs the following steps iteratively:

1. Calculate the determinant for each submatrix $M_{i,i}$ for $i\in \{1,\dots ,N\}$.
2. Keep $M_{i,i}$ for which the determinant is largest.
3. If $\det (M_{i,i}) > T$, return $M_{i,i}$ and all nodes that have been removed, otherwise continue with step 1.

Once the dimensionality of the bivariate network has been reduced according to the above-described algorithm, the steps to retrieve the multivariate network from the reduced bivariate network are the same as described in Mader et al.^5^ In short, these steps consist of the following:

Starting from the reduced bivariate network, represented here by the correlation matrix $\varrho =M_{i,i}$, the matrix is inverted

1 $$\begin{aligned} g = \rho ^{-1} \end{aligned}$$

and normalized ${\tilde{\pi }} = \tilde{h} \cdot g \cdot \tilde{h}$. The normalization matrix $\tilde{h}$ is a diagonal matrix with $\tilde{h}_{ii} = g_{ii}^{-1/2}, i=1,\dots ,n$. The off-diagonal elements of ${\tilde{\pi }}$ are multiplied by $-1$ to arrive at the partial correlation matrix $\pi$, representing the reduced multivariate network.

The previously removed nodes are then added back to the reconstructed multivariate network. Their weighted links to the other nodes in the network remain unchanged from those of the bivariate network.

The method’s steps described above are illustrated for an example network in Fig. 1c–e. Here, the threshold is set to $T=0.1$. The determinant of the matrix representation *M* of the bivariate network shown in (c) is $\det (M)=0.07$. The algorithm finds that the submatrix $M_{1,1}$ has the largest determinant with $\det (M_{1,1})=0.77$. The reduced network is inverted, and the previously removed node $n_1$ is added to the reconstructed multivariate network. Note that in the example given here, node $n_1$ and $A_1$ are swapped in the layout of the reconstructed multivariate network in Fig. 1. This is to emphasise that node $n_1$ has been removed before the matrix inversion. When comparing the reconstructed multivariate network in (e) to the actual multivariate network in (a) as described in Sec. “Construction of multi- and bivariate networks”, the networks need to be topologically equivalent in order to be comparable. Because node $A_1$ has been used in the matrix inversion, node $A_1$ is included in comparison with node $n_1$ of the true multivariate network. Therefore, node $A_1$ is shown in the position of node $n_1$ in the reconstructed multivariate network shown. The exchange of $A_1$ and $n_1$ is immaterial for the conclusion drawn as it is impossible to distinguish the role of these two nodes given their high correlation.

### Construction of multi- and bivariate networks

To be able to evaluate the algorithm introduced in ﻿Sec. “Method steps”, the reconstructed multivariate network is compared to the true multivariate network in a simulation study. For this comparison, we create multivariate networks and use them to construct the bivariate networks that would be observed in a correlation analysis. The mathematical framework is described in the following:

The correlation matrix representing the bivariate network is constructed from a multivariate network, represented by the partial correlation matrix $\pi$. The correlation matrix is derived through the following steps^5^: First, the off-diagonal elements *R* are separated from the partial correlation matrix

2 $$\begin{aligned} \pi = 1 + R. \end{aligned}$$

Changing the sign of the matrix *R* and applying a matrix inversion yields

3 $$\begin{aligned} \tilde{\rho } = (1-R)^{-1}. \end{aligned}$$

The corresponding correlation matrix is obtained through normalizing $\tilde{\rho }$ by dividing each entry of $\tilde{\rho }$ by $s_i s_j$, with $s_i = \tilde{\rho }_{ii}^{1/2}$.

The correlation matrix

4 $$\begin{aligned} \rho = R R^T, \end{aligned}$$

is decomposed using the *Cholesky decomposition* with *R* a lower triangular matrix and $R^T$ its transpose. The matrix *R* is multiplied to *n* uncorrelated random variables of length $L_{RV}$ drawn from a Gaussian distribution. This transforms the uncorrelated Gaussian random variables to random variables that have correlation matrix $\rho$.

Extra nodes are randomly added to the network by adding random variables that duplicate any of the existing random variables with added noise of different standard deviations. Each newly added random variable has a high correlation with the original random variable if the standard deviation of added noise is low but the nodes remain linearly independent. The final correlation matrix is estimated based on the full set of random variables; it is of higher dimensionality than the original one with a small but non-zero determinant.

This procedure is illustrated for the example network discussed above in Fig. 1a–c. The node $A_1$ results from adding a random variable equal to $n_1$ with added noise. The constructed bivariate network in (c) is used as a starting point for reconstructing the multivariate network shown in (c)-(e) and discussed in ﻿Sec. “Method steps”.

### Assessing the reconstructed networks

Constructing the bivariate network based on the multivariate network as described in ﻿Sec. “Construction of multi- and bivariate networks” makes it possible to compare the reconstructed to the original multivariate network. Both the dimensionality-reduction and the direct-inversion method can be compared by directly assessing the similarity of the reconstructed network and the original one.

Compared to the original, the reconstructed multivariate networks have a higher dimensionality due to the added nodes. To be able to compare the networks, they need to have the same dimensionality. For the direct-inversion method, the added nodes are removed from the reconstructed network, and a reduced network consisting only of the original nodes is compared to the original multivariate network. For the dimensionality-reduction method, the nodes included in the matrix inversion are kept and compared to the original network. If an original node had been removed previously, the corresponding highly correlated node that is included in the matrix inversion is compared to the original node.

The networks are compared using two network properties characterising the network as a whole and not individual nodes, the absolute difference and the small-worldness of both networks^19^. The absolute difference is calculated by subtracting the matrix representation of both networks and taking the sum over the absolute values of all differences. The small-worldness is measured by a real value $\sigma$ as described in ﻿Sec. “Small-world network”.

Figure 1e–h shows the difference between the actual and reconstructed multivariate network for the example presented in Fig. 1a–e. The reconstructed multivariate network based on dimensionality-reduction in (e) and direct-inversion method in (g) are compared to the actual multivariate network in (a). In (g) the subnetwork consisting of nodes $n_1$ - $n_4$ for the direct-inversion method and in (f) the subnetwork consisting of the nodes $A_1, n_2, n_3, n_4$ for the dimensionality-reduction method are compared to the actual network. The node $A_1$ is used instead of $n_1$ because $A_1$ was included in the matrix inversion while node $n_1$ was removed, see above. Note that the algorithm removes node $n_1$ instead of $A_1$ purely based on a higher determinant of the submatrix and independent of which node has been added in the construction of the bivariate network.

In the case of multiple added nodes, highly correlated with the same original node, the algorithm sometimes finds a higher determinant by removing the original node followed by another node of the network that has not been used to construct additional nodes. In this case, the nodes of the reconstructed multivariate network do not match the nodes of the original network. Two nodes correspond to the same original node, and one node in the original network does not have a corresponding node in the reconstructed network. To still compare the two networks, we compare one of the nodes corresponding to the same original node with the original node that has no corresponding node in the reconstructed network. This choice is taken arbitrarily to be able to compare the networks and has no further implication.

### Small-world network

A small-world is an essential characteristic for a type of network that combines a strong local clustering defined by a high average clustering coefficient typical for regular networks with the short characteristic path length typically found in random networks through long-range interactions^6,19–21^.

The average clustering coefficient for positive $C^{+}$ or negative $C^{-}$, respectively, weights are measured by

5 $$\begin{aligned} C^{\pm } = {\left\{ \begin{array}{ll} \frac{1}{N} \sum _{i} \frac{1}{d^{\pm }_i(d^{\pm }_i-1)}\sum _{jk}w_{ij}^{\pm }w_{jk}^{\pm }w_{ki}^{\pm } &{},\, d_i^{\pm }>1\\ 0 &{},\, \text {otherwise} \end{array}\right. } \end{aligned}$$

with $w_{ij}^{\pm }$ being the positive/negative weighted link between nodes *i* and *j* and $d_{i}^{\pm }=\sum _j w_{ij}^{\pm }$ the weighted positive/negative degree of node *i*. The characteristic path length is calculated through

6 $$\begin{aligned} L = \frac{2}{N(N+1)}\sum _{i=1, j< i}^N l_{ij}, \end{aligned}$$

with $l_{ij}$ being the shortest distance between nodes $n_i$ and $n_j$^19^.

Since the measures used throughout this study are correlations, the network weights must first be transformed into distances to calculate $l_{ij}$. To be able to interpret weights as distances, the weighted links of networks used in the analysis of the small-worldness are restricted to positive correlations $w_{ij}\in [0,1]$ in the simulation. The correlations can be transformed to distances using a logarithmic transformation $\tilde{l}_{ij}=-\log (w_{ij})$, transforming small correlations to large distances and large correlations to small distances. The shortest distance between each pair of nodes is obtained by taking the minimum of all possible ways from $n_i$ to $n_j$^22^.

To make both clustering coefficient and characteristic path length interpretable independent of *N*, they are normalized by calculating the average of both measurements $C_r$ and $L_r$ over 1000 random networks. The random networks are restricted to the same constraints as the multivariate networks generated in the simulation. A single measure for the small-worldness of a network can be obtained by combining *C* and *L* into

7 $$\begin{aligned} \sigma = \frac{C/C_r}{L/L_r}. \end{aligned}$$

A network is said to have small-world characteristics if $\sigma >1$^23^.

### Simulation setup

Each simulation is conducted by randomly generating 100 symmetric and positive definite multivariate networks of size *N*, with $N=5$ in ﻿Sec. “Finding the right threshold” and for each $N\in \{5, 10, 15\}$ in ﻿Sec. “Difference of weights compared between methods”, “Small-World” and “Numerical differences”. The number of links are Gaussian distributed, rounded to the nearest integer, with a mean of $N(\log (N))$ and weights representing correlation coefficients. In general, the correlation coefficients are randomly selected from the interval $[-1,1]$. For reasons of interpretability, the correlation coefficients are restricted to positive values only when analysing the small-world characteristics of the networks in ﻿Sec. “Small-world”. The multivariate networks are inverted according to ﻿Sec. “Construction of multi- and bivariate networks” to obtain the initial bivariate networks.

The determinants of both, original multi- and bivariate network have to exceed a threshold, arbitrarily chosen to be 0.1 in all simulations. This ensures that correlations between added and original nodes are higher than in the original bivariate network, lowering the determinant of the bivariate network with added nodes.

For each original bivariate network, ten bivariate networks with higher dimensionality and lower determinant are constructed by randomly adding highly correlated nodes as described in ﻿Sec. “Construction of multi- and bivariate networks”.

### Ill-conditioned matrix due to added random processes

A small determinant of a bivariate network can also be caused by simply adding uncorrelated nodes to the network. To illustrate this, we add Gaussian noise as new nodes to the bivariate network shown in Fig. 1b with a length of $L_{RV}=100$. The bivariate network is decomposed in the same way as described in ﻿Sec. “Construction of multi- and bivariate networks” using the Cholesky decomposition.

### Algorithm implementation

All code used for the implementation of the algorithm and simulation study has been implemented in Python 3.9. The customised functions use the Python modules system 3.9.1, numpy 1.22.2 and pandas 1.4.0. Generating the networks in the simulation study requires additionally the packages SciPy 1.8.0 and scikit-learn 1.0. The network analyses described in ﻿Sec. “Small-world network” make additionally use of the packages networkx 2.7.1 and bctpy 0.5.0.

The illustrations in Figs. 1 and 2 are created in Inkscape 1.1, the Figs. 3, 4, 5, 6, 7, 8 are plotted using the Python packages matplotlib 3.5.0 and seaborn 0.11.2.

The code used in this study is freely available from the authors on reasonable request.

### Ethics declarations

The study was approved for North American investigator sites and UK NHS sites by the Quorum Review IRB and West Midlands-Coventry and Warwickshire Research Ethics Committee, respectively. The EudraCT number is 2014-002156-61. The subject gave informed consent to have their data that are analysed in the supplementary information, recorded, analysed, and published anonymously. All experiments were performed in accordance with relevant guidelines and regulations.

## Results

This section evaluates the dimensionality-reduction method and compares it to the direct inversion, described and analysed in Mader et al.^5^. The setup for the simulations is described in “Simulation setup”.

In ﻿Sec. “Finding the right threshold” the reconstructed multivariate networks based on the dimensionality-reduction method are compared to the original networks for different thresholds. The change of the difference between the determinant of the original bivariate network and bivariate network with added nodes when iteratively removing nodes are compared as well.

In ﻿Sec. “Difference of weights compared between methods” the two methods of direct inversion and dimensionality reduction are compared by calculating the absolute difference and the small-worldness of the networks in ﻿Sec. “Small-world”. ﻿Section “Numerical differences” analyses numerical differences between original and reconstructed multivariate caused by the Cholesky decomposition.

### Finding the right threshold

The dimensionality-reduction method removes nodes from the bivariate network based on a threshold for the determinant. If the threshold is set too small, the algorithm does not remove any nodes; it is equal to the direct-inversion method. If the threshold is chosen too large, the algorithm will remove nodes with low correlations to the rest of the network. If these nodes contribute essential information to the network topology, the reconstructed multivariate network deviates from the actual multivariate network.

For the simulations conducted here, the actual multivariate networks are known so that the correct threshold can be determined: For a threshold set in the correct range, the algorithm reduces the dimensionality of the bivariate network with added nodes to the size of the original network. It removes either the previously added nodes or the original nodes that share a high correlation with the added nodes. A threshold chosen too small does not remove enough nodes, including high correlations in the matrix inversion. A threshold chosen too large reduces the dimensionality further than the original network, removing nodes that have not been added or duplicated and contribute accurate direct links to the network.

**Figure 2:**
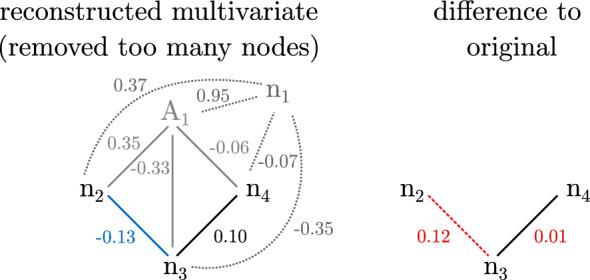
Left: Dimensionality-reduction method applied to bivariate network as shown in Fig. 1c–e with threshold $T=0.8$. The determinant of the reduced bivariate network after removing node $A_1$ is 0.77. The algorithm continues by removing node $n_1$, increasing the determinant to 0.97, exceeding the threshold. Right: Difference between reduced networks and the corresponding nodes of the original multivariate $n_2$ - $n_4$. The removal of node $n_1$ creates a false link between nodes $n_2$ and $n_3$.

An example for a threshold chosen too large for the network discussed in Fig. 1 is shown in Fig. 2. The threshold is increased from $T=0.1$ (see ﻿Sec. “Method steps”) to $T=0.8$. This causes the algorithm to remove node $A_1$ in addition to $n_1$. A comparison of the weights of the inverted nodes shows that node $n_2$ and $n_3$ are now connected by a link that did not exist in the original network. This is not the case for the correctly chosen threshold as shown in Fig. 1f where the weights of the reconstructed network are highly similar to those of the original network.

**Figure 3:**
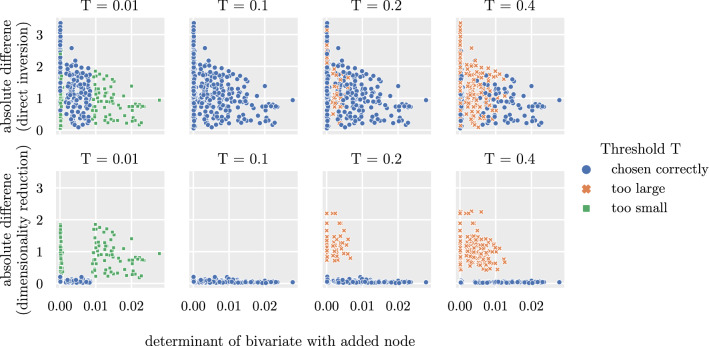
Comparison of absolute difference versus determinant of the bivariate network with added nodes for direct-inversion (top) and different thresholds *T* for the dimensionality-reduction method (bottom). The networks fall into three categories: Networks for which the threshold is chosen correctly are shown in blue circles. If the threshold is too small, the networks are depicted in green squares and if the threshold is too large they are colored in orange crosses. Note that the color code is used for both applied methods for comparison; however, the threshold has an effect only on the dimensionality-reduction method.

Figure 3 shows the absolute difference between reconstructed and original network in a simulation study with a network size $N=5$ for four different thresholds. Shown are both the direct-inversion and the dimensionality-reduction method; the threshold affects only the latter.

A threshold $T=0.01$ is too low for some of the networks; highly correlated nodes are included in the matrix inversion resulting in a large absolute difference between reconstructed and original multivariate network. At the threshold $T=0.01$, the absolute difference jumps from low to large values. The bivariate networks near this threshold are the original bivariate networks with one added node. If the added node lowers the determinant below *T*, the node is removed, and the multivariate network is reconstructed correctly. If the determinant is above *T*, the node does not get removed, and the method is identical to the direct-inversion method. This can be seen by comparing the absolute difference between both methods: The absolute difference between the original and reconstructed multivariate network is the same for all networks to the right of $T=0.01$. A second jump can be seen near zero. Networks that are shown here have two added nodes, lowering the determinant close to zero. For the networks shown in green, the algorithm removes some but not all of the added nodes. After removing the first node, the determinant is larger than the threshold, and one added node remains in the network - the reconstructed multivariate deviates from the original.

For $T=0.1$ the algorithm removes only the previously added nodes or their corresponding highly correlated original nodes, and the absolute difference is small for all generated networks.

If *T* is increased further to $T=0.2$, the algorithm removes more nodes than added to the original bivariate network causing a large absolute difference between the original and reconstructed network comparable with the direct-inversion method.

For $T=0.8$ the threshold is too large for all generated networks, leading the algorithm to remove more nodes than previously added in all networks. Based on these results, the threshold is set for all following simulations to $T=0.1$.

**Figure 4:**
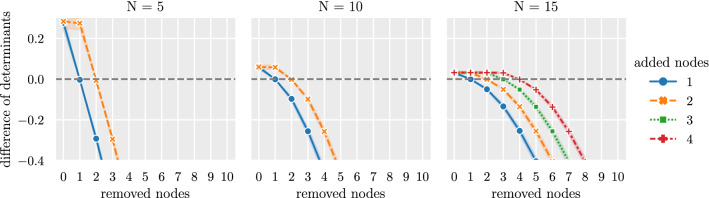
Change of determinant when iteratively removing nodes from the bivariate network. Simulated are 100 multivariate networks for three different network sizes $N\in \{5,10,15\}$. Bivariate networks with added highly correlated nodes are constructed by adding 1 and 2 nodes for network sizes $N\in \{5,10\}$ and adding 1,2,3 and 4 nodes for $N=15$. Shown is the difference between the determinant of the original bivariate network and the determinant of the bivariate network with added nodes. If the difference is positive, not all previously added nodes are removed from the network. A difference of zero indicates that the number of removed nodes is equal to the number of previously added nodes. A negative difference between the determinants indicates that more nodes are removed than previously added to the network. The shaded area is a $95\%$ confidence-interval over each set of 100 generated networks.

Figure 4 shows how the determinant of the network changes when the algorithm iteratively removes nodes. Shown is the difference between the determinant of the original bivariate network and the bivariate network with added nodes. For a positive difference, the determinant of the original network is greater than the one of the reconstructed network. When the determinant of the reconstructed network exceeds the determinant of the original network, the difference becomes negative. The difference remains positive and changes only in small steps until all previously added nodes or the corresponding highly correlated actual nodes have been removed. With the removal of the last previously added node the determinant of the reconstructed network increases, hence the difference decreases rapidly for all further removed nodes. This sudden change allows to find a correct threshold. Nodes should be removed as long as the determinant changes little. As soon as the determinant increases greatly for the first time, it is likely that the correct threshold is found.

### Difference of weights compared between methods

**Figure 5:**
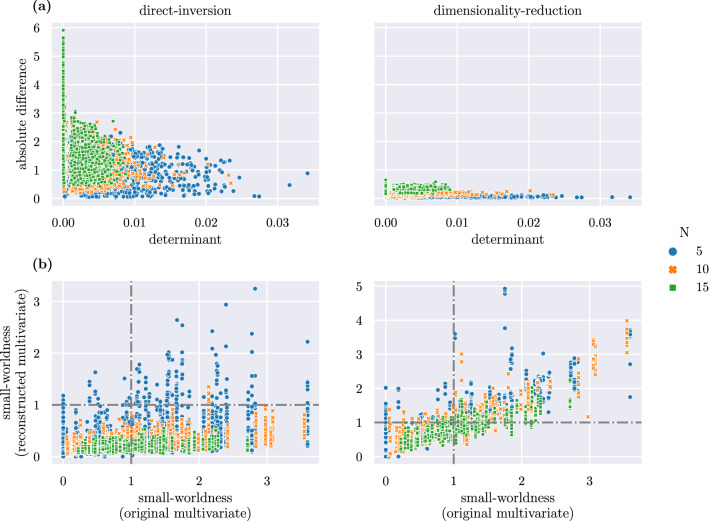
Generated 100 multivariate networks of size *N*, with $N\in \{5, 10, 15\}$. For each corresponding bivariate generated 10 bivariate networks with 1-3 added nodes highly correlated to one of the existing nodes. Compared are the direct-inversion (left) and dimensionality-reduction method (right). (**a**) Shown is the absolute difference between original and reconstructed multivariate in relation to the determinant of the bivariate with added nodes. (**b**) Shown is the small-worldness $\sigma$ of the reconstructed multivariate networks according to both methods in comparison with the small-worldness of the original network. A value of $\sigma >1$ indicates a small-world network. This threshold is indicated by a horizontal and vertical line at $\sigma =1$.

Figure 5a shows the absolute difference between reconstructed and original multivariate matrix over the determinant of the bivariate network with added nodes. The absolute difference between original and directly inverted matrix is shown on the left and on the right for the reconstructed matrix using dimensionality reduction. The difference of the dimensionality-reduction method is an order of magnitude smaller than for the direct inversion. This shows that the weights of the reconstructed multivariate networks based on dimensionality reduction deviate only in very small amounts from the original networks.

The difference is negatively correlated with the determinant of the correlation matrices with added nodes. The smaller the determinant, i.e. the larger the correlation between some of the nodes, the more the reconstructed partial correlation matrix deviates from the original.

The absolute difference scales with $N^2$. The absolute difference is summed over the absolute value of all weighted links; hence small deviations between the weights and numerical uncertainties are summed up in the absolute difference, scaling with the number of links $N^2$. Numerical uncertainties that are introduced to the network because of the construction of the bivariate network using the Cholesky decomposition are discussed in ﻿Sec. “Numerical differences”.

### Small-world

**Figure 6:**
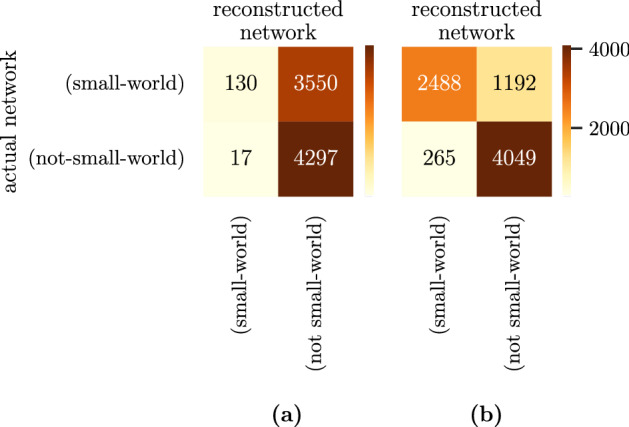
Confusion matrices showing the small-worldness of original and reconstructed multivariate network shown in Fig. 5b. (**a**) The direct-inversion method falsely classifies most small-world networks as random networks. (**b**) The dimensionality-reduction method correctly classifies a majority of networks.

Figure 5b shows a comparison of the small-worldness $\sigma$ calculated for the reconstructed multivariate networks with direct inversion (left) and dimensionality reduction (right). Figure 6 shows that the direct-inversion method falsely classifies almost all networks as non-small-world. The dimensionality-reduction method classifies the majority of networks correctly.

### Numerical differences

**Figure 7:**
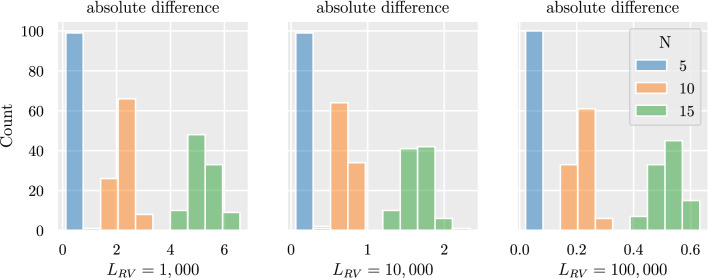
Numerical differences between original and reconstructed multivariate network based on Cholesky decomposition. Shown is the distribution of absolute difference for 100 generated multivariate networks for each $N\in \{5,10,15\}$. The absolute difference is smaller for increasing length of random variables $L_{RV}$ used to reconstruct the bivariate networks - the absolute difference scales with the number of links in the network $N^2$.

This section analyses the numerical differences that occur due to the reconstruction of the random variables using the Cholesky decomposition as described in ﻿Sec. “Construction of multi- and bivariate networks”. For this simulation, 100 multivariate networks are generated for each $N\in \{5,10,15\}$. The networks are inverted and the Cholesky decomposition is used to construct the corresponding random variables. They are correlated again directly, without adding any nodes. The resulting correlation matrix of random variables deviates from the original bivariate network due to the finite length of the random variables. The larger the size of the random variable, the closer the correlation of random variables is to the original bivariate network. Figure 7 shows the absolute difference between original multivariate network and reconstructed based on the random variables for different lengths $L_{RV}$ of 1000, 10,000 and 100,000. The absolute difference becomes smaller with larger $L_{RV}$. The absolute difference also scales with $N^2$, as explained in ﻿Sec. “Difference of weights compared between methods”.

### Additional Gaussian noise processes can lower the determinant

**Figure 8:**
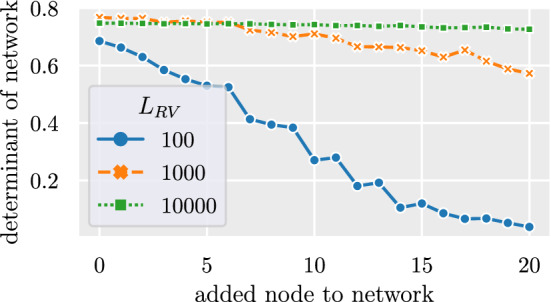
Change of determinant when adding Gaussian noise processes to the network. The bivariate network in Fig. 1b is decomposed into the corresponding random variables using the Cholesky decomposition. The random variables have a length of $L_{RV}\in \{100,1,000,10,000\}$. Normally distributed random variables are iteratively added to the network. The determinant decreases with added random variables. The rate of decrease dependents on the size of the random variables.

Figure 8 shows the determinant of the bivariate network shown in Fig. 1b with up to 20 added nodes as described in ﻿Sec. “Ill-conditioned matrix due to added random processes”. The determinant decreases with each added node depending on the size of the random variable. This shows that a low determinant does not have to be the result of highly correlated random variables but can be caused by independent random processes. However, for a comparable determinant of the network to the bivariate network with a single highly correlated node shown in Fig. 1c, it requires several added random processes that are much smaller compared to the added node.

## Discussion

We have shown that existing methods are not able to reconstruct the multivariate network from a bivariate network when highly correlated nodes are present. We propose an alternative method that first reduces the dimensionality based on the determinant of the network before inverting it. We compared the new method with existing methods and showed that it correctly reconstructs the underlying topology where existing methods fail. Section “Finding the right threshold” has shown the impact of differently chosen thresholds for the determinant for the dimensionality-reduction method to the absolute difference between original and reconstructed multivariate network. It shows the importance of the threshold in the reconstruction of the multivariate network. If the threshold is chosen too low, highly correlated nodes do not get removed, and the method becomes equal to and hence suffers from the same challenges as the direct-inversion method. If the threshold is chosen too large, too many nodes are removed, and the reconstructed multivariate network deviates from the original network. Removed nodes that do not share high correlations with the rest of the network contribute essential information about direct links that are lost if the nodes get removed. This is illustrated for an example in Fig. 2. Removing one of the nodes $n_1$ or $A_1$, as shown in Fig. 1e, leads to a very accurate reconstruction of the multivariate network. The high correlation between both nodes of $r=0.95$ leads them to contribute similar, almost redundant, information to the network. However, removing both nodes removes information that is essential to infer some of the true direct links in the network: In the original network, there exists no direct link between node $n_2$ and $n_3$ but an indirect link through node $n_4$ and either of node $n_1$ or $A_1$. If both nodes $n_1$ and $A_1$ are removed, this path becomes inaccessible, and the algorithm falsely identifies the indirect link between node $n_2$ and $n_3$ as a direct connection. Figure 3 shows the impact of the threshold quantitatively. Exemplarily shown for four different thresholds, it shows the importance of the chosen threshold to reconstruct the multivariate network correctly. In the simulation studies conducted here, the correct threshold can be inferred from the construction process of the bivariate networks. Because the nodes added to the original bivariate network share high correlations with some of the original nodes, the determinant decreases after adding the nodes. Setting the threshold to the determinant of the original bivariate network will cause the algorithm to remove precisely the added or corresponding original nodes from the network.

When applied to networks where the original bivariate network is unknown, the threshold must be found experimentally. A suggestion for how to infer the correct threshold if the true multivariate network is unknown can be derived from Fig. 4. The figure shows that the determinant of the network changes in small steps until all but one previously added node have been removed. For each simulated network and independent of the network size or number of added nodes, the correct threshold corresponds to the first time that the determinant of the network increases significantly. This indicates that all nodes with high correlations have been merged, and any further removal of nodes will cause a loss of topologically important information.

If applied to other bivariate networks that are not constructed in the way described here, the decision for setting the threshold could be made based on the rate of change of the determinant: If the transition of the rate of change corresponds to the one shown in Fig. 4, the decision for the threshold is unambiguous. If the rate of change follows a smoother transition, this method suggests a range for the threshold. The rate of change of the determinant at the chosen threshold can indicate how likely it is for the reconstructed network to deviate from the actual multivariate network. This translates into an approach how a good threshold can be derived: By removing nodes from the network and consecutively quantifying the impact this removal has on the determinant suggests a threshold that should be used, for instance by investigating rapid changes in this relation. It should be noted that this is a trade-off between accuracy and resulting network size. An example of an application to real data can be found in the supplementary information. The dimensionality-reduction method is applied to a correlation network based on an electroencephalography (EEG) dataset where the actual underlying topology is unknown. Directly inverting the correlation network leads to a spurious partial correlation matrix, dominated by highly correlated nodes. The dimensionality-reduction method correctly identifies and removes highly correlated nodes and thereby reconstructs a robust partial correlation matrix of the reduced network.

We have directly compared the dimensionality-reduction method to the direct inversion in ﻿Sec. “Difference of weights compared between methods”. The differences are shown in Fig. 5a for bivariate networks with a low determinant. Here, the dimensionality-reduction method leads to an order of magnitude smaller absolute difference compared to the direct inversion. The absolute difference is negatively correlated with the determinant of the network when the bivariate network is directly inverted. This shows that the reconstructed network based on the direct-inversion method becomes closer to the true multivariate network if the determinant of the bivariate network is larger. If there are no highly correlated nodes in the bivariate network, the dimensionality-reduction method does not remove any nodes, provided the threshold for the determinant is chosen correctly. In this case, the method is equivalent to the direct-inversion method and the actual multivariate network can be correctly reconstructed without removing any nodes. If a node needs to be removed, this inherently implies that information is lost. The higher the correlation, the more information is preserved. If in an application an information loss is unacceptable, or needs to be minimised below a certain threshold, dedicated simulation studies are highly recommended to investigate the extent of the problem. We note, however, that a compromise needs to be found between acceptable information loss and being able to calculate a valid partial correlation matrix. If no information loss is acceptable, the multivariate counterpart to the bivariate network cannot be calculated. The true underlying network structure remains hidden in this case.

We have further investigated the impact of this finding on the small-worldness of the reconstructed multivariate network in ﻿Sec. “Small-world”. The direct-inversion method falsely classified most small-world networks as not small-world while the dimensionality-reduction method successfully reconstructs a multivariate network of the same property as the original network. These findings show that not only the weights of the reconstructed multivariate network deviate when a bivariate network with low determinant is directly inverted, but also fundamental topological properties such as the small-worldness of the network are lost.

An alternative approach to find an approximate solution for the inverse problem of ill-conditioned matrices is to apply regularisation methods such as ridge regression and Lasso^17,18^. In general, these techniques introduce a penalty term or Lagrange parameter that regularises the matrix such that a matrix inversion becomes numerically possible. The inverted matrix could then serve as an approximation of the partial correlation matrix.

We have investigated how ridge regression compares to the direct-inversion and dimensionality-reduction methods, using the network shown in Fig. 1 as an example. We found that the absolute difference of the reconstructed multivariate network using ridge-regression is larger for all tested regularisation parameters, suggesting that these methods reconstruct the underlying topology better. It would be interesting to investigate how such approximating techniques handle high correlation in comparison with our new method in greater detail. Such a comparison is beyond the scope of this manuscript, as we focused on introducing a novel approach that does not rely on such regularisation approaches.

Section “Numerical differences” analyses the numerical differences between the original and reconstructed multivariate network that arise because of the finite size of the random variables used in the Cholesky decomposition. Figure 7 shows how the absolute difference between original and reconstructed multivariate network scales with the size $L_{RV}$ of the random variables. From the comparison of the numerical differences shown in the right plot for $L_{RV}=100,000$ with the absolute difference shown in the right graph in Fig. 5a, it can be seen that the deviations between the reconstructed and the original multivariate network are explained mainly by these numerical differences.

The focus was to reconstruct multivariate from bivariate networks with highly correlated nodes and resulting small determinant. We have shown in ﻿Sec. “Additional Gaussian noise processes can lower the determinant” that high correlations are not the only possible reason for a low determinant. We have shown exemplarily at the bivariate network used in Fig. 1b how adding random processes to random variables lead to a decreasing determinant of the network. The dimensionality-reduction method would likely fail to reconstruct the multivariate network correctly because the small determinant is not caused by highly correlated nodes but by completely independent random processes. Figure 8 shows that the influence of random processes on the determinant is gradual. It also shows an effect only for random variables with relatively few data points. For independent random processes to have a similar effect on the determinant as highly correlated nodes, it requires many added random processes compared to the original network size. If these additional uncorrelated processes have a small number of observed data points they should be filtered out before the dimensionality-reduction method is applied. This is possible because bivariate analysis will provide the correct result that those processes are not linked to the network of interest. It is worth noting that this is a particular important step if the number of these uncorrelated processes is large compared to the number of data points.

## Conclusion

Previous studies have shown that inverting the bivariate network can successfully reconstruct the multivariate network^5^. However, this requires the corresponding correlation matrix to have a full rank. Here we have investigated the case of highly correlated nodes that result in an ill-conditioned matrix in the bivariate network. We have been able to explain that the direct inversion fails to reconstruct the multivariate network correctly. We have proposed an alternative method that first reduces the dimensionality of the bivariate network based on a threshold for the determinant before inverting the correlation matrix. This method can reconstruct the multivariate structure correctly. It also correctly identifies a network as small-world while the direct inversion fails. When using the direct-inversion method, the absolute difference becomes smaller with an increasing determinant of the bivariate network, suggesting that the method leads to a correctly reconstructed multivariate network if the determinant of the network is large enough. We have shown that the threshold of the determinant for the bivariate network is crucial for a correct reconstruction of the multivariate network when using the dimensionality-reduction method. Setting the threshold too low leaves high correlations in the network that distort the retrieved direct links; setting the threshold too large leads to a removal of links that are essential for reconstructing the actual topology of the network. For networks where the corresponding multivariate network is unknown, we suggested a method to determine the correct threshold based on the change of rate of the determinants of the reduced networks. We show the successful application of the method to real data with unknown underlying topology in the supplementary information.

In addition, the dimensionality-reduction method is applicable in cases of linearly dependent nodes in which a direct inversion is not only numerically but also mathematically impossible. It becomes possible to reconstruct part of the multivariate structure, allowing valuable insight into the actual network topology, which would not be accessible otherwise.

## Supplementary Information

Supplementary Information.

## Data Availability

The participant of the EEG-study that is conducted in the supplementary information did not agree for their data to be shared publicly, so supporting data is not available.
