# Supplementary material for: Inferring the underlying multivariate structure from bivariate networks with highly correlated nodes
Source: Sci Rep. 2022 Jul 21;12:12486. doi: 10.1038/s41598-022-16296-y (PMC9304421; doi:10.1038/s41598-022-16296-y)
Supplement: Supplementary file 1 — Supplementary Information. [file 41598_2022_16296_MOESM1_ESM.pdf]

Inferring the underlying multivariate structure from bivariate networks with highly correlated nodes: Supplementary Information

Philipp Loske[1,*] **and Bjoern O. Schelter**[2,3]

[1]Aberdeen Biomedical Imaging Center, University of Aberdeen, Foresterhill, Aberdeen, UK
[2]TauRx Therapeutics Ltd., Aberdeen, UK
[3]Institute for Complex Systems and Mathematical Biology, University of Aberdeen, Aberdeen, UK
[*]p.loske.18@abdn.ac.uk

1 Regularisation methods using the example of ridge-regression

Here, we compare the direct-inversion and our novel dimensionality-reduction method with regularisation methods[1,2] using ridge-regression[3]. When inverting a matrix is either not possible or the matrix is ill-conditioned, regularisation methods provide an approximate solution to the inverse-problem that is numerically invertible. Ridge regression introduces a constraint to the bivariate network, called penalty or Lagrange paramenter λ. The correlation matrix ρ in Eq. (1) is approximated by the regularised matrix

$$\rho_r = \rho + \lambda I, \tag{S1}$$

where I is the identity matrix. The penalty parameter λ is typically in the range of 0 to 1, where $\lambda = 0$ corresponds to the unregularised correlation matrix. The diagonal elements of the correlation matrix ρ are always equal to 1, the penalty parameter takes the role of *loading* these diagonal elements to values larger than 1. Increasing λ reduces the correlation between nodes and makes the matrix numerically invertible.

1.1 Results

Using the example of the bivariate network with highly correlated nodes in Fig. 1 (c), we show how the network can be regularised and the multivariate network reconstructed. The reconstructed network is compared with the reconstructed networks based on the standard approach of direct inversion and our novel approach that reduces the dimensionality of the bivariate network first. The results are shown in Fig. S1, with the networks shown in matrix form to illustrate how the penalty parameter changes the diagonal values of the correlation matrix. We found the lowest absolute difference for $\lambda = 0.2$. The example shows that ridge-regression does not improve the reconstructed network compared to the direct-inversion method.

A detailed simulation study could help determine whether there are cases where ridge-regression or alternative regularisation methods improve the results of the direct-inversion method. Our example shows that our novel method outperforms both alternatives in accurately reconstructing the multivariate network.

2 Dimensionality-reduction method applied to EEG data

In this section, we apply our new approach to real-world data. While in this application the true underlying topology is unknown, this example helps to demonstrate the working of the approach. The dataset is a resting-state electroencephalogram (EEG) taken from a healthy 80-year-old male subject. It consists of 21 time series representing the 21 electrodes of the standard 10:20 EEG recording system. Each consists of 100 seconds of data sampled at 200 Hz.

2.1 Results

The correlation matrix of all 21 electrodes is shown in Fig. S3 (a). It represents a fully connected network with in particular two very highly correlated nodes (A1, A2). If applying the matrix inversion directly to this ill-conditioned correlation matrix to obtain the partial correlations, these two highly correlated nodes dominant the multivariate matrix as expected (Fig. S3 (a)). Given the theoretical work in the manuscript, this is driven by the very small positive determinant of $< 10^{-30}$. Applying our novel approach, initially the nodes A1 and A2 are removed (see Fig. S3 (b)). The partial correlation matrix is significantly improved compared to the one obtained by simple inversion of the original correlation matrix. This clearly demonstrates that removal of these two nodes improves the network analysis.

As indicated, the underlying correct network is not know such that the process of node removal should arguably continue. Following Sec. 3.1, we first analyse how the determinant changes with the number of removed nodes. The results can be seen in

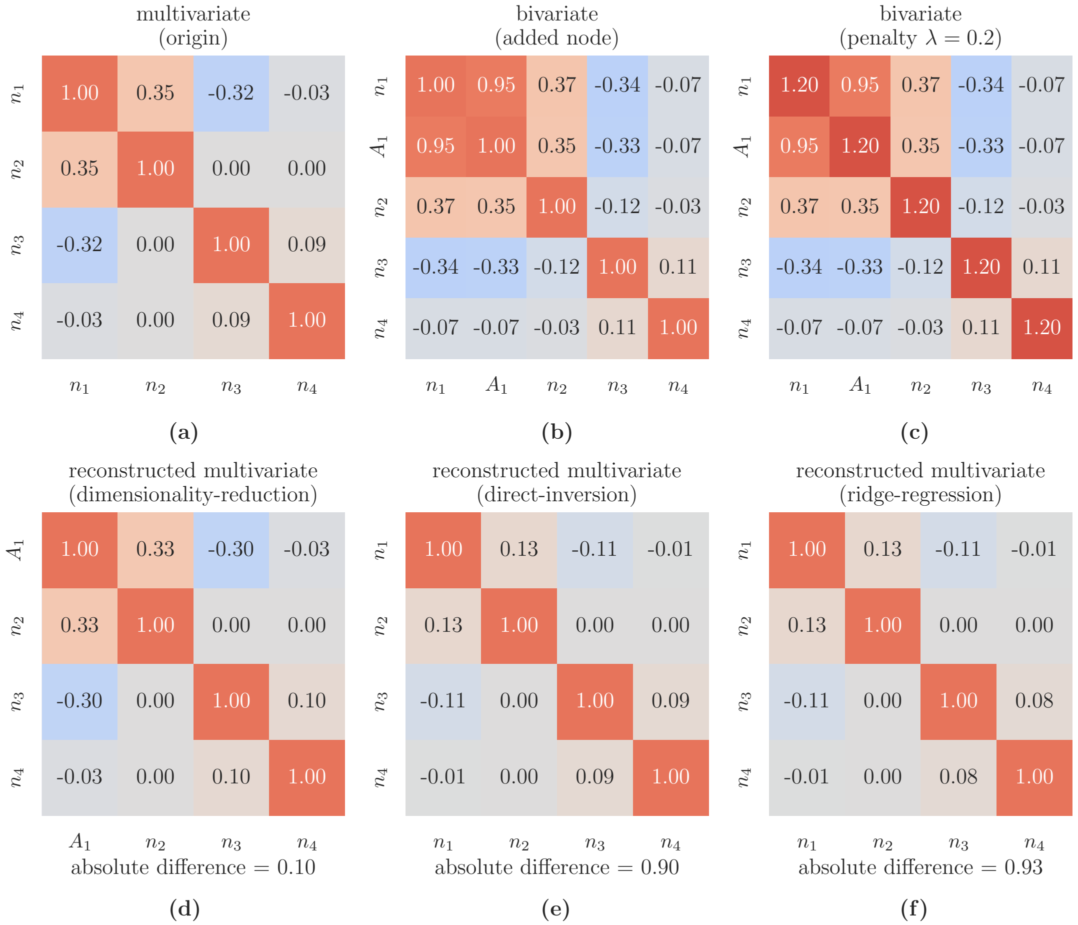

Figure S1. Comparison of ridge-regression, direct-inversion and dimensionality-reduction method applied to the network shown in Fig. 1. The networks are presented in matrix form to show how ridge-regression changes the diagonal elements of the matrix. For better readability the matrices in **(d)-(f)** show only the four nodes used to calculate the absolute difference. **(a)** The partial correlation matrix representing the original multivariate network. **(b)** The correlation matrix corresponding to the bivariate network with added highly correlated node A_1 with an determinant of 0.07. **(c)** The regularised correlation matrix using ridge-regression with a penalty parameter $\lambda = 0.2$. The determinant increases to 0.42. **(d)** The reconstructed multivariate network according to the dimensionality-reduction method. **(e)** The reconstructed multivariate network according to the direct-inversion method. **(f)** The reconstructed multivariate network based on ridge-regression. The absolute difference to the original multivariate network in (a) are shown below the matrices.

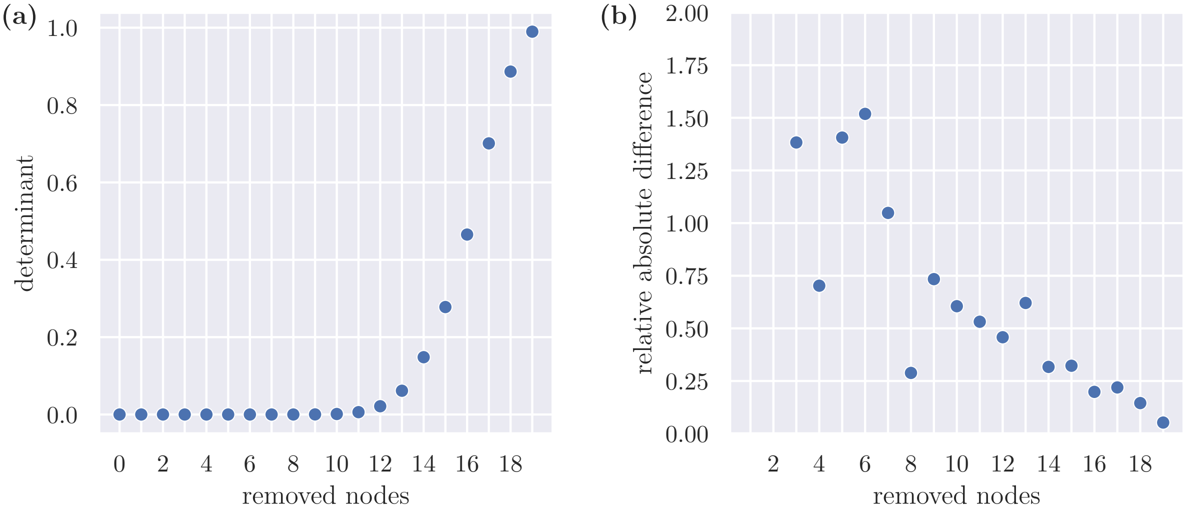

Figure S2. **(a)** Determinant of reduced bivariate measure depending on the number of removed nodes. **(b)** The relative absolute difference of the reconstructed multivariate after removing n nodes with the reconstructed multivariate network after removing $n-1$ nodes from the bivariate network. The relative absolute difference is the absolute difference divided by the size of the network.

Fig. S2 (a). The determinant increases from near zero for the original bivariate network to one when there are only two nodes left in the network. As explained in Sec. 3.1, the rate of change of the determinant is an indication of a correct threshold. Visually, the determinant changes notably at around 10 removed nodes. The corresponding correlation and partial correlation matrices are shown in Fig. S3 (c).

The absolute difference between reconstructed and actual multivariate network cannot be calculated because the actual topology of the correlation network is unknown. Instead, we introduce a supporting measure to determine the correct threshold for the determinant: the relative absolute difference between each reduced bivariate matrix after removing n nodes and the matrix after removing $n-1$ nodes. The relative absolute difference is the absolute difference as explained in Sec. 2.3, divided by the network size n. This measure is used to compare how much the reconstructed multivariate network changes at each step during the removal of nodes. Figure S2 (b) shows that this relative difference becomes smaller with each node removed. The behaviour stabilises at around 10 nodes, which again suggests that this is a very good approximation of the correct number of nodes to be removed.

References

1. Krämer, N., Schäfer, J. & Boulesteix, A.-L. Regularized estimation of large-scale gene association networks using graphical gaussian models. *BMC bioinformatics* **10**, 1–24 (2009).

2. Epskamp, S. & Fried, E. I. A tutorial on regularized partial correlation networks. *Psychol. methods* **23**, 617 (2018).

3. Hoerl, A. E. & Kennard, R. W. Ridge regression: Biased estimation for nonorthogonal problems. *Technometrics* **12**, 55–67 (1970).

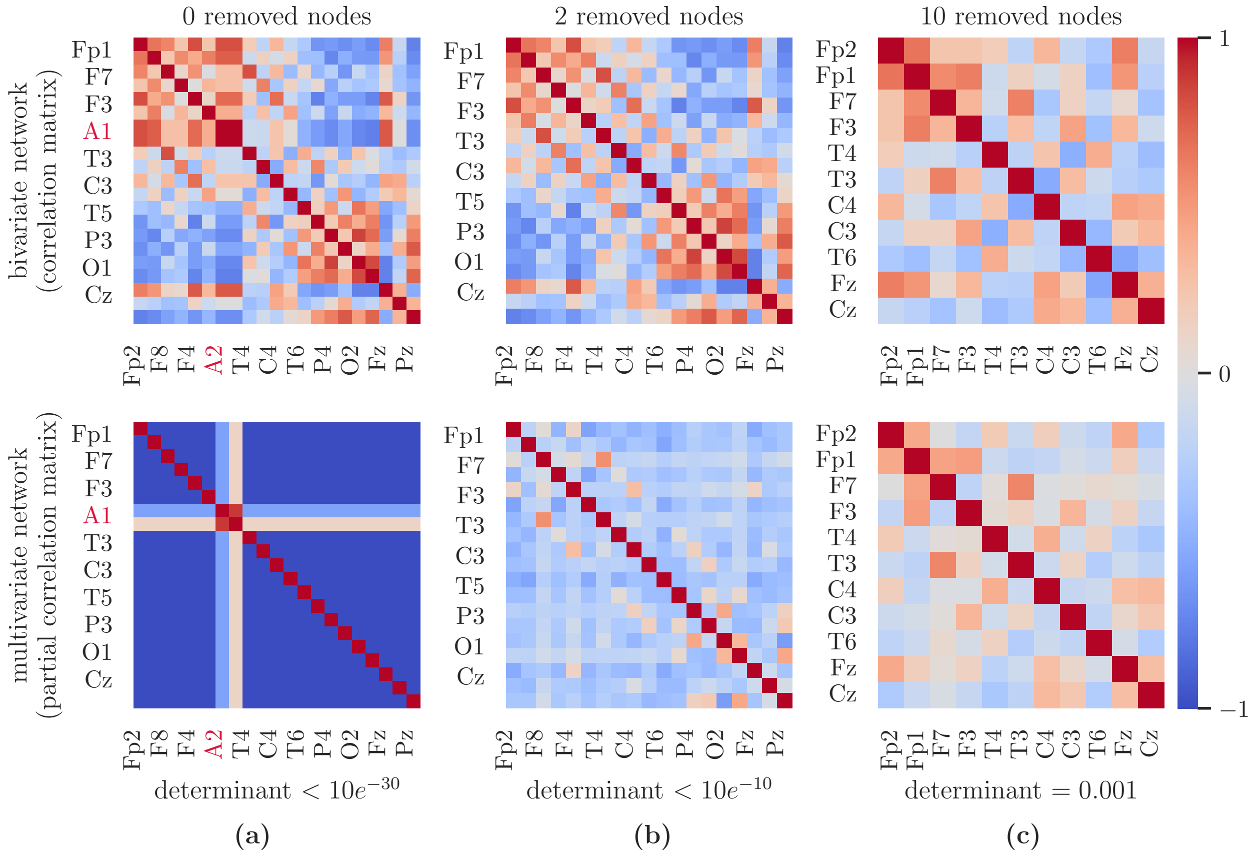

Figure S3. Correlation and partial correlation matrices of an EEG dataset. The partial correlation matrices are derived using the dimensionality-reduction method applied to the bivariate network. Shown are pairs of correlation and partial correlation matrix for different number of removed nodes. The number of removed nodes is shown above and the determinant of the reduced bivariate network below each pair of matrices. Note that the labels of the columns and rows are identical. The columns and rows in (a) and (b) are each labelled alternately with only half of the labels for better readability. **(a)** Original bivariate and multivariate network. The correlation matrix is numerically invertible but the resulting reconstructed multivariate network is not useful. The highlighted nodes A1 and A2 are the first two nodes that are removed in (b). **(b)** Reduced bivariate and reconstructed multivariate network after the removal of node A1 and A2. The partial correlation matrix is significantly improved. **(c)** Reduced bivariate and reconstructed multivariate network for 10 removed nodes (removed: F8, F4, A2, A1, T5, P4, P3, O2, O1, Pz). The relative absolute difference is small (see Fig. S2 (b)) and the determinant significantly increased.
